# Extraction Methods Affect the Structure of Goji (*Lycium barbarum*) Polysaccharides

**DOI:** 10.3390/molecules25040936

**Published:** 2020-02-19

**Authors:** Shengyi Zhou, Atikur Rahman, Junhui Li, Chaoyang Wei, Jianle Chen, Robert J. Linhardt, Xingqian Ye, Shiguo Chen

**Affiliations:** 1 Department of Food Science and Nutrition, Zhejiang Key Laboratory for Agro-Food Processing, Fuli Institute of Food Science, Zhejiang R & D Center for Food Technology and Equipment, Zhejiang University, Hangzhou 310058, China; zjuchowchow@163.com (S.Z.); atik.fpe@hstu.ac.bd (A.R.); 18511581374@163.com (J.L.); weichaoyang2012@163.com (C.W.); 3090100118@zju.edu.cn (J.C.); 2 Department of Chemistry and Chemical Biology, Center for Biotechnology and Interdisciplinary Studies, Rensselaer Polytechnic Institute, Troy, NY 12180, USA; linhar@rpi.edu

**Keywords:** extraction methods, structural characterization, rhamnogalacturonan I, homogalacturonan

## Abstract

Polysaccharides are considered to be the most important active substances in Goji. However, the structure of polysaccharides varies according to the extraction methods applied, and the solution used to prepare Goji polysaccharides (LBPs) were limited. Thus, it is important to clarify the connection between extraction methods and structure of Goji polysaccharide. In view of the complex composition of cell wall polysaccharides and the various forms of interaction, different extraction methods will release different parts of the cell wall. The present study compared the effects of different extraction methods, which have been used to prepare different types of plant cell wall polysaccharides based on various sources, on the structure of cell-wall polysaccharides from Goji, by the single separate use of hot water, hydrochloric acid (0.4%) and sodium hydroxide (0.6%), at both high and low temperatures. Meanwhile, in order to explore the limitations of single extraction, sequential extraction methods were applied. Structural analysis including monosaccharide analysis, GPC-MALLS, AFM and ^1^H-NMR suggested the persistence of more extensively branched rhamnogalacturonan I (RG-I) domains in the procedures involving low-temperature-alkali, while procedures prepared by high-temperature-acid contains more homogalacturonan (HG) regions and results in the removal of a substantial part of the side chain, specifically the arabinan. A kind of acidic heteropolysaccharide was obtained by hot water extraction. SEC-MALLS and AFM confirmed large-size polymers with branched morphologies in alkali-extracted polysaccharides. Our results provide new insight into the extraction of Goji polysaccharides, which differ from the hot water extraction used by traditional Chinese medicine.

## 1. **Introduction**

The cell wall polysaccharides of plant contain mostly pectin, lignin, hemicellulose and cellulose, with pectin being the main active polysaccharide. According to the structure of the molecular backbone and side chains, pectic polysaccharides can be categorized into four major groups: homogalacturonan (HG), rhamnogalacturonan I (RG-I), rhamnogalacturonan II (RG-II) and xylogalacturonan (XG) [1]. HG (about 65% of commercial pectin) is the most abundant and structurally found to have a linear chain of (1-4)-linked α-d-GalAp units [2], while the backbone of RG-I is composed of alternating rhamnose and galacturonic acid residues (1-2 and 1-4 linked) [3]. RG-I is present in the fruits, roots, stems and leaves of plants, linking with cellulose and hemicellulose, as well as cell wall proteins [4].

Different extraction methods obtain different parts of the cell wall and result in differences in the structure of the extracted polysaccharides [5]. Hot acid solution has been widely used to prepare commercial pectin [6], and hot water extraction is the most common method for preparing traditional Chinese medicine polysaccharides, such as ganoderma [7], mushroom [8] and *Cordyceps sinensis* [9]. The study of Khodaei [10] confirmed that HG-domain-pectin could be prepared by hot acid extraction. Zykwinska [11] proved that pectins easily extracted with alkali at 40 °C and 65 °C were enriched in homo- and rhamnogalacturonans with arabinan side chains in limited amounts. The research results of Zhang [12] showed that the isolation of mandarin peel pectic polysaccharides enriched in RG-I could be evaluated through a sequential extraction method, consisting of acid followed by alkaline hydrolysis at room temperature. It seems that different extracted solution and conditions will result in different types of polysaccharides, but the extraction rules are still inconclusive.

Goji (*Lycium barbarum*) is a solanaceous defoliated shrub that is found in arid and semi-arid regions of Northwestern China, Southeastern Europe and in Mediterranean countries [13]. Polysaccharides have been reported to be among the most important active, potent and well-reported substances prepared from goji in addition to polyphenols, flavonoids, vitamins, carotenoids, saponins and polypeptides. These polysaccharides have many potential bioactive functions as antioxidants [14,15,16], immunomodulatory agents [17], anticancer agents [18,19], neuroprotective agents [20,21] and anti-aging agents [22]. 

Hot water extraction, the most commonly used method to extract goji polysaccharides (LBPs), rely on a solid-liquid ratio ranging from 1:10 to 1:35, extraction time from 2 to 7 h and extraction temperature from 70 to 100 °C [23]. As modern novel approaches can improve extraction efficiency, in some cases, hot water extraction is assisted by microwave and ultrasound. Yang [24] evaluated the yields and properties of LBPs extracted from goji by different methods including hot water (HWE 100 °C), ultrasonic water (UWE 30–40 °C), subcritical water (SWE 110 °C), and ultrasound-enhanced subcritical water extraction (USWE 110 °C). The total LBP yield was the highest from USWE (14% *w/w*) and the lowest from HWE (7.6%). Muatasim [25] confirmed that the yield of crude polysaccharides increased by 73.41% using the dual-frequency ultrasound extraction compared to traditional hot water extraction. Besides for that, mixed enzymes, such as pectinase, protease and cellulase, have been commonly used to extracting LBPs [26,27].

Although LBPs have been widely studied, most studies focused on optimizing yield, while the solution used to prepare LBPs were limited and few studies have been conducted on the mechanism of the extracted process. The mechanism of various extraction methods needs to be better understood. Studying the dissolution mechanism of LBP to improve the extraction rate represents a key research direction in the future. In addition, the influence of the extraction system on both recovery and polysaccharide structure needs to be clarified.

Therefore, this study aimed to extract water-soluble LBPs from goji (*L. barbarum*) using different extraction solvents and conditions, which have been used to extract different types of plant cell wall polysaccharides based on various sources, including hot water, 0.4% hydrochloric acid and 0.6% sodium hydroxide both at high and low temperature. The LBPs extracted with different solvents were then also characterized based on their physiochemical properties such as yield, total sugar, total protein, monosaccharide composition, weight-average molecular weight and preliminary structural features. The effects of extraction methods on structure of goji polysaccharides were investigated and compared.

## 2. Results and Discussion

### 2.1. Physicochemical Properties and Monosaccharide Composition Analysis

The yield, total sugar, total protein and monosaccharide composition of the extracted fractions are presented in Table 1. On dry weight basis, AL_H_, A_H_ and AL_L_ had high yields of 4.18%, 2.5% and 2.46%, respectively. Less than 2.0% yields of A_L_, W_H_AL_L_, A_L_AL_L_, W_H_, W_H_AL_H_ and A_H_AL_H_ were obtained. Chang [28] showed that water is the least effective solvent for extracting pectin, which was confirmed in this study. Temperature also showed a strong influence on the yield. The lower temperature caused less loosening of the cell-wall structure and a smaller extent of pectin solubilization [29]. The protein contents were high in the variety of ‘Ningqi-7’ relatively, and glutamic acid, aspartic acid, leucine were the main components in protein of all samples.

The alkali-treated samples had a low galacturonic acid (GalA) content compared to acid and water extraction approaches (AL_L_: 14.53%; A_L_: 28.24%; AL_H_: 6.34%; A_H_: 23.67%), which may due to alkali condition caused the degradation of HG region by β-elimination and oxidative peeling reactions [11]. The ratio of (Ara + Gal)/Rha was calculated as an estimate of the relative importance of the neutral side-chains to the backbone. As the Rha/GalA and (Ara + Gal)/Rha ratio and for AL_L_ was 0.527 and 7.77 respectively, suggesting that AL_L_ consists mainly of RG-I structure with highly branched morphologies [30]. The Rha/GalA ratio for AL_L_ was much higher than that of the commercial citrus pectin and apple pomace pectin, which were characterized by the predominance of long HG regions [31]. Indeed, to preserve RG-I regions including neutral sugar side chains and induce an important degradation of HG regions, alkaline treatment appeared the most appropriate [32]. 

Acid extractions that are well known to lead to high pectin solubilization, however, such treatment can result in the degradation of important side chains [33]. Taking the GalA contents and (Ara + Gal)/Rha ratio for acidic-extracted polysaccharides into account, A_L_ and A_H_ both contain more HG regions and have lost a substantial part of their side chains, specifically arabinan. The neutral side branches of A_L_AL_L_ or A_H_AL_H_ were less than those obtained in the single-alkali-extracted samples. The residue, which had been treated with acid, contained few neutral side chains for further extraction. 

**Figure 1:**
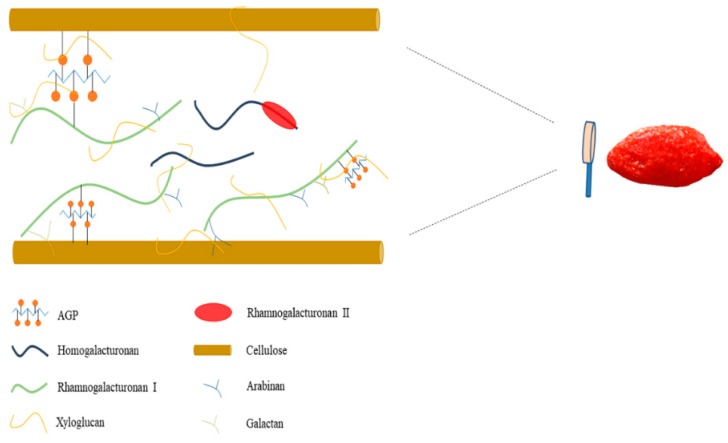
Microscopic structural components of the goji cell wall.

The protein content of goji polysaccharides extracted by alkali was higher than that extracted by acid or water. Since the alkali were applied to second steps of all sequential extractions, the protein contents of sequential-extracted goji polysaccharides were also high. The trend in protein content was consistent with the trend in neutral sugar (arabinose and galactose) content, as alkali-extracted polysaccharides possessed more branched side chains while acid degraded them. In view of the fact that arabinogalactan-protein (AGP) is an important glycoconjugate presented in LBPs, a large part of the neutral sugar branches of RG-I should be tightly bound to protein in goji pectic polysaccharides [34]. The molecular weight of AGP in goji varies from 24 to 920 kDa [35], so it was hypothesized that, even if the side chains are degraded during extraction, the glycoconjugate will be co-precipitated together with polysaccharides and will not be all lost on dialysis because of their large molecular weight. According to this conjecture, a schematic diagram of the goji cell wall is shown in Figure 1.

As the temperature increases, the more side branches are extracted and more are cut off at the same time [33]. Alkaline extraction at high temperature caused more significant degradation of arabinose than galactose, in agreement with previously published data [11]. As the ratio of (Ara + Gal)/Rha was 4.9 in A_H_, and 5.82 in A_L_, indicating the side branches of A_H_ was less than A_L_. From continuous extractions, the neutral portion released by hot alkali after hot-acid-extraction, was far less than the hot-alkali-extracted sample (A_H_AL_H_: 17.73% Ara; AL_H_: 29.17% Ara). The missing neutral sugar has been pre-released by the hot acid. 

The RG-I content of A_L_AL_L_ is higher than A_L_, and the HG content of these two samples was equivalent, suggesting that the RG-I extracted by low-temperature-alkali were difficult to be extracted by low-temperature-acid. Considering the diversity of the structure and combination of pectin, the RG-I extracted by low-temperature-alkali may be closely linked with cellulose and hemicellulose in the cell wall, while low-temperature-acid could only extract the part that was loosely bound and easy to be released.

The Rha/GalA of W_H_AL_L_ was greater than that of AL_L_, indicating the polysaccharides extracted by hot water were non-RG-I pectin. Similarly, the HG content of W_H_AL_L_ was also greater than that of AL_L_, suggesting that the polysaccharides extracted by hot water were non-HG pectin. Judging from this, the substance obtained by hot water extraction was a kind of acidic heteropolysaccharide that was free in the plant cell wall. 

### 2.2. Homogeneity, Molecular Weight and Conformation of Lbps

**Figure 2:**
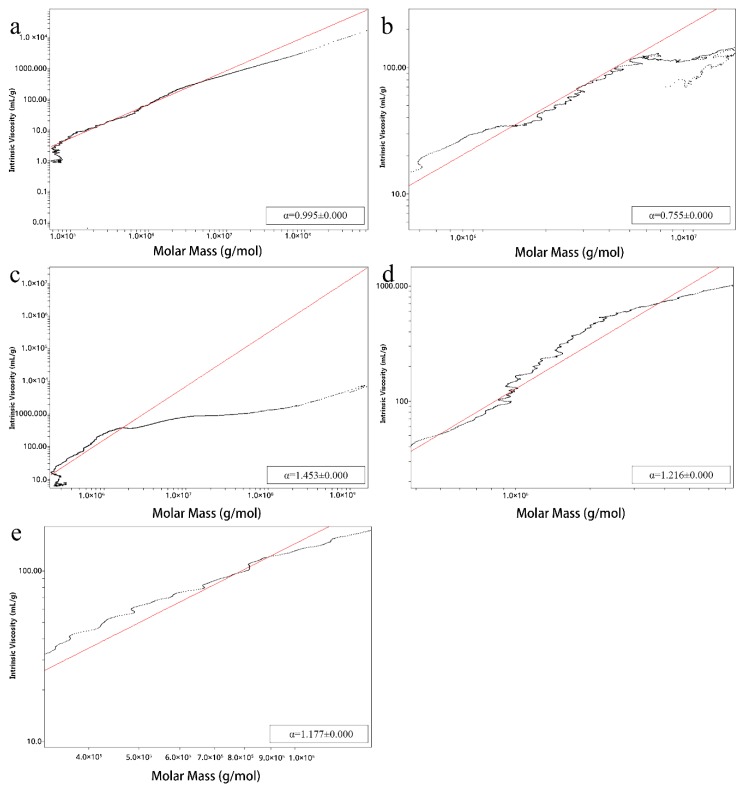
Mark-Houwink-Sakurada equation of (**a**) AL_H_, (**b**) AL_L_, (**c**) A_H_, (**d**) A_L_ and (**e**) W_H._

Relative values were calculated using Astra 6.1 (Wyatt Technologies, Santa Barbara, CA, USA) to obtain more accurate information about the molecular size of the LBPs. Mark–Houwink–Sakurada plots of AL_L_, A_L_, W_H_, AL_H_ and A_H_ are shown in Figure 2. All samples contained two main components, which may represent neutral and acidic fragments in LBPs [36], and molecules that are relatively large (Table 2). The mass-average molar mass (Mw) of acid-extracted polysaccharides were significantly smaller than alkali-extracted ones (AL_L_: 7162 kDa, 1582 kDa; A_L_: 2334 kDa, 717.4 kDa; AL_H_: 223.1 kDa, 26.32 kDa; A_H_: 199.2 kDa, 3.954 kDa), which may result from alkali-extracted samples retaining more neutral sugars and glycoprotein side chains, and so the molecular weight is slightly larger than that of acidic-extracted ones. Similarly, as high temperatures lead to more pieces of the polysaccharide chains [29], polysaccharides extracted at low temperature have a larger Mw than the high temperature-treated samples. 

The molecular radius of gyration (Rz) of the acid-extracted polysaccharides was larger than that of the alkali-extracted polysaccharides. This can be explained if polysaccharides containing higher Ara have a larger molecular weight but smaller Rz than those containing higher GalA [37]. The molecular weight of AL_L_ and A_L_ are much higher than the previously published LBP-4a (34kDa), LbGp3 (50—60kDa) and LBP1 (925kDa) [35,38,39], which may be due to the better preservation of side chains during the low temperature extraction in the present study.

The molecular weight of The Mark–Houwink–Sakurada equation ([η] = KMα) was applied to calculate the chain conformations of AL_L_, A_L_, W_H_, AL_H_ and A_H_ in 0.15 M NaCl solution. Alkali-extracted samples were predicted to be branched, since the exponent (α) of Mark–Houwink–Sakurada equations for AL_L_, A_L_, W_H_, AL_H_ and A_H_ was 0.755, 1.216, 1.177, 0.995 and 1.453, respectively (Figure 2), indicating that alkali-extracted polysaccharides exhibit irregular coil conformation while acid- or hot water-extracted polysaccharides have a rigid chain conformation [33].

### 2.3. Nanostructure Analysis

**Figure 3:**
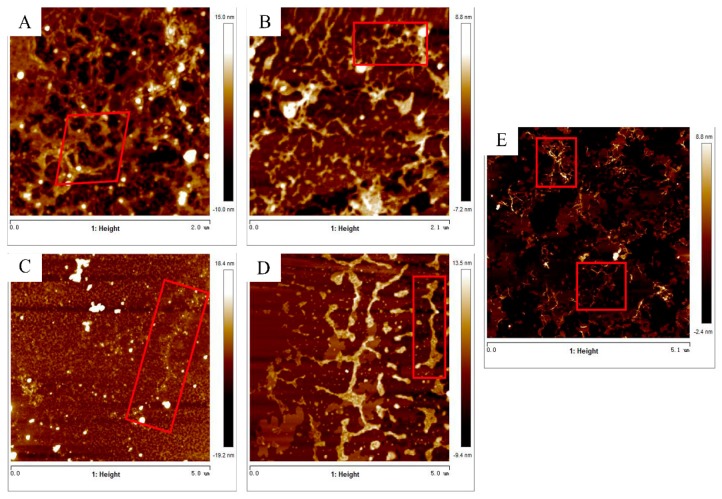
Representative topographical AFM images of (**A**) AL_H_, (**B**) AL_L_, (**C**) A_H_, (**D**) A_L_ and (**E**) W_H_. Three main forms (p, br and mbr) could be observed in alkali-extracted Goji polysaccharides, which possess more side chains and they’re intertwined. The acid-extracted Goji polysaccharide has fewer branches and the side chains mainly exhibit two forms (p, br). The chain morphology of W_H_ was disordered.

AFM imaging was done for nanostructural characterization of single extracted samples. Four major nanostructures were observed in samples (Figure 3): linear single-fragment structure (ls), single-branched structure (br), multi-branched structure (mbr) and polymer (p) structure. A number of long-chain branched structures are attached to the linear backbone in alkali-extracted polysaccharides, consistent with the slope of the Mark–Houwink–Sakurada equation fitting line. These branched structures are intertwined to form a complex polymer, leading to three main forms (p, br and mbr). The acid-extracted Goji polysaccharide has fewer branches, as they were cut down more branched side chains. As the temperature increases, the neutral part of the polymer is degraded and becomes shorter, which may due to high temperature can reduce the abundance of arabinose and galactose [40]. 

### 2.4. FT-IR Analysis

**Figure 4:**
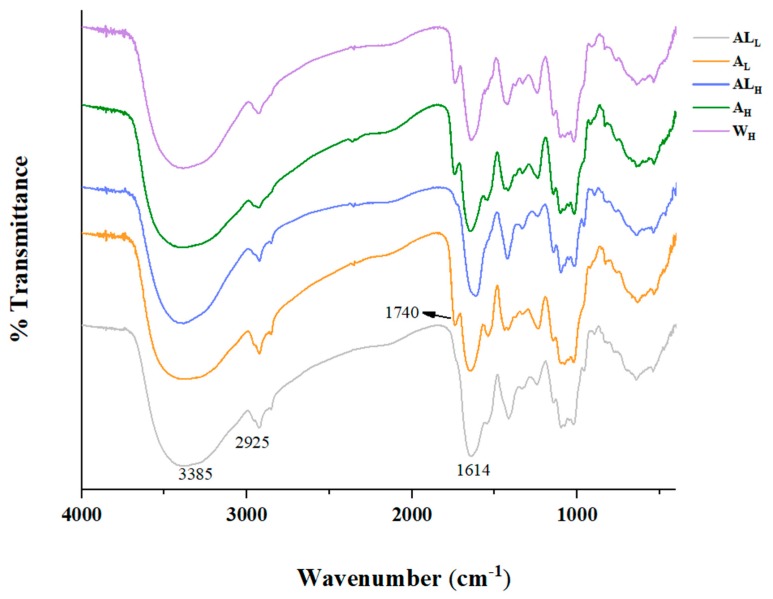
FT-IR spectra of A_H_, AL_H_, A_L_, AL_L_ and W_H_.

The FT-IR spectra (Figure 4) showed characteristic absorption peaks of single extracted samples. The existence of two peaks absorbing at 1740 and 1614 cm^−1^ clearly indicated the presence of COOCH_3_ and COO^−^ in pectin GalA [41]. Since the pH of the cell wall material suspension is neutral and the pKa of polyGalA is 3.38, it is clear that all non-esterified carboxyl groups are in the form of carboxylate ions [42]. Therefore, the sum of the areas of the bands at 1740 and 1614 cm^−1^ was proportional to the degree of esterification. The calculation formula is DM (%) = A1740/(A1740 + A1614) × 100% [43]. When coming to analyze the infrared spectra of AL_H_ and AL_L_, there were hardly any peaks at 1740 cm^−1^, indicating that alkaline extraction leads to de-esterification of the pectic polysaccharides, consistent with previous studies [12,44]. The alkali extraction procedure may include the dissolving out of pectic polysaccharides and simultaneously result in the de-esterification of the pectic polysaccharides. 

At 950–800 cm^−1^ an absorption peak for glucose, mannose and galactose is observed, which is caused by the C–H variable angle vibration of β-d-pyranose. This shows certain differences in monosaccharide composition among all samples, which is consistent with the monosaccharide analysis. 

### 2.5. NMR Analysis

**Figure 5:**
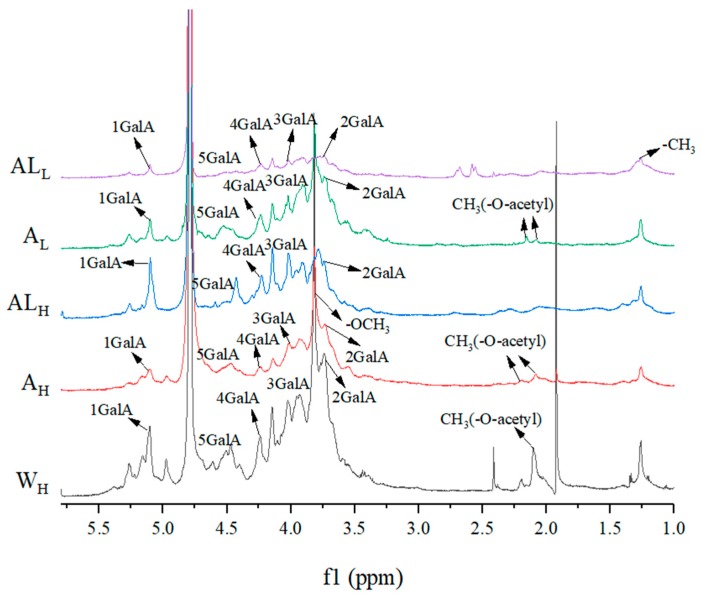
^1^H-NMR spectra of A_H_, AL_H_, A_L_, AL_L_ and W_H_.

The structural features of single extracted samples were further analyzed by ^1^H-NMR (Figure 5). The anomeric proton signals at relatively high field, 3.5–5.1 ppm, suggest that the type of glycosidic bond is primarily β-glycosidic in all of the LBPs, in agreement with the studies of Yuan [45]. The intense signal at 3.82 ppm in the sample was generated by a methoxy group (–OCH_3_) attached to the carboxyl terminus of GalA. The two signals near 2.0 ppm are the acetyl signals attached to the O-2 and O-3 sites of GalA, respectively. In a previous report Liu [36], hot water-extracted p-LBP showed –CH_3_ groups of α-Gal*p*A on O-2/3. However, in the current study no protons of methyl ester groups or acetyl groups in AL_H_ and AL_L_ were detected, further indicating that alkaline-treatment polysaccharides were non-esterified. In the anomeric region, the signals near 5.16 ppm were attributed to the H-1 of different types of Ara. The signals for Ara in the alkali-extracted samples spectrum were larger than those in acidic-extracted ones, which is consistent with the results of monosaccharide compositional analysis. The signal near 1.26 ppm was the methyl signal linked to O-2 and O-2,4 of l-rhamnose. Both the acid-extracted and the alkali-extracted polysaccharides had signals here, indicating there were pectic polysaccharides in AL_L,H_ and A_L,H_, with different proportions of RG-I regions.

## 3. Materials and Methods

### 3.1. Materials

The fruits of Goji (*Lycium barbarum* cv. ‘Ningqi-7’) were collected from Xinjiang Ougan Agricultural Technology Co., Ltd’s goji cultivation base, east of Wushitara township in the Xinjiang Autonomous Region, China. Analytical grade chemicals were obtained from Sinopharm Chemical Reagent Co. Ltd (Shanghai, China) unless noted otherwise. A549 cells were kindly donated by the Zhejiang Academy of Medical Sciences.

### 3.2. Extraction of LBPs

**Figure 6:**
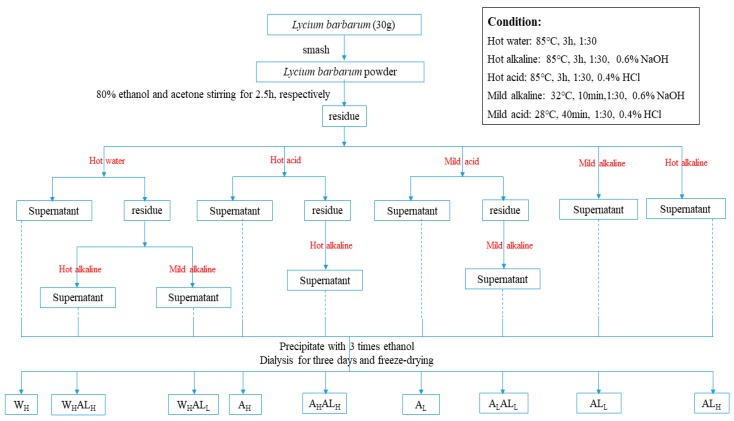
Process flowchart for extraction of LBPs.

The dried ripe fruits of goji (1.0 kg) were first ground into powder and then immersed in acetone and 80% ethanol for 3 h, followed by drying, resulting in pre-treated goji powder. For the extraction of pectic polysaccharides, the powder (1:30, *w/v*) was used for single and sequential extractions following the scheme in Figure 6. Single extractions were performed using hot water, 0.4% hydrochloric acid and 0.6% NaOH, respectively. Sequential water-alkali extraction was performed by adding 0.6% NaOH to the hot water-extracted residues. The same procedure was repeated in sequential acid-alkali extraction in which 0.6% NaOH was added to the acid-extracted residue. Acid- and alkali-related extractions were all performed at both low and high temperatures. High-temperature extractions were performed at 85 °C for 3 h. Low-temperature acid extractions were performed at 28 °C for 40 min with simultaneous stirring, while low-temperature alkaline extractions were performed at 32 °C only for 10 min with stirring as well. Each suspension was filtered and the residues were washed with 70% ethanol until the filtrate showed a negative reaction by the phenol-sulfuric acid test [46]. The extraction conditions are based on those used in previous studies [12]. After acid extraction, the pH of the resulting suspension was adjusted to 3–4, while that of suspension extracted by alkali was adjusted to 6–7. After filtration and centrifugation, three volumes of 95% ethanol were added to the concentrated retentate for precipitation at 4 °C for 24 h. Finally, in every case, after precipitation, the resulting precipitates were collected and washed alternately with absolute ethanol and acetone, three-times. These washed precipitates were collected and dialyzed against water using a dialysis membrane (MWCO 10000 Da) for 2 days and finally freeze-dried. The crude polysaccharide was obtained after ethanol precipitation and vacuum freeze-drying.

### 3.3. Determination of Total Sugar, Protein Content and Amino Acid Composition

Total sugar content was measured by the phenol-sulfuric acid method with D-glucose as standard [47]. The Bradford assay, with bovine serum albumin as standard [48], was used to determine the protein content of the LBPs. The amino acid composition was analyzed by HPLC. Briefly, 7 mg dry samples were dissolved in 6 mL 4 mol/L hydrochloric acid solution, and digested at 110 °C for 22 h. After cooling, the solution was diluted and 2 mL of the supernatant was evaporated to dryness. Finally, 1 mL 0.2 mol/L hydrochloric acid solution was added, and the amino acid composition was measured after filtration.

### 3.4. Analysis of Monosaccharide Composition by HPLC

Monosaccharide composition was analyzed by the 1-phenyl-3-methyl-5-pyrazolone (PMP)-HPLC method described previously by Strydom [49], with some modifications. Briefly, samples (2–3 mg/mL) were first hydrolysed with 2M TFA at 110 °C for 8 h in an ampoule. After being fully hydrolyzed, the excess acid was removed by a stream of nitrogen by adding 200 µL methanol and the same step was repeated three times followed by neutralization with 0.1M NaOH. For further derivatization, the hydrolyzates were then dissolved with 450 μL of 0.3M sodium hydroxide and 450 μL PMP solution (0.5M, in methanol) and then the mixtures were allowed to react at 70 °C for 30 min. The resulting solutions were neutralized by 0.3M hydrochloric acid and the solutions obtained were extracted three times using the same volume of chloroform to remove excess reagent. The upper phase was filtered through a 0.22 μm membrane, and 1 mL of the resulting solution was injected for analysis. A Waters e2695 separation module (Waters, Milford, MA, USA) with a Zorbax Eclipse XDB-C18 column (250 mm × 4.6 mm, 5 μm, Agilent, Santa Clara, CA, USA) was used to perform HPLC analysis at 25 °C. The elution rate was 1 mL/min. Detection was carried out with a 2489 UV/Vis Detector (Waters) at 250 nm.

### 3.5. Determination of Molecular Weight and Conformation

The weight-average molecular weight (Mw), number-average molecular weight (Mn = ∑NM/∑N, N: molecular weight of each component; M: the number of moles of each component), polydispersity (Mw/Mn) and chain conformation of LBPs were estimated by high-performance size exclusion chromatography using an instrument equipped with a multi-angle laser light-scattering system with a refractive index detector (HPSEC-MALLS-RI, Wyatt Dawn Heleos-II, Wyatt Technology, Santa Barbara, CA, USA) according to the method described by Arakawa [50], with some modifications. Isocratic elution with 0.20M NaCl solution at a flow rate of 0.5 mL/min was performed on a Shodex SB-804 HQ and SB-806 HQ (Showa Denko KK, Tokyo, Japan) at 25 °C. Sodium chloride solution (0.20M NaCl) was used as the mobile phase at a flow rate of 0.5 mL/min. The injection volume was 50 µL and running time was 100 min. ASTRA software (version 7.1.2, Wyatt Technology) was used to obtain the data.

### 3.6. Atomic Force Microscopy (AFM)

Molecular shapes of LBPs were acquired by AFM. LBPs (1 mg/mL) were first dissolved in ultrapure water (Milli-Q level) and then incubated at 80 °C for 2 h with continuous stirring. Sodium dodecyl sulfate (SDS) was selected as a hydrogen bond cleavage agent to disperse the entangled polymer chain. After cooling to room temperature, the solution was diluted with SDS until the solution contained an equal mass of LBPs and SDS at a concentration of 1 μg/mL. The diluted solution was then stirred for 24 h at room temperature and filtered through a 0.22 μm filter. A 10 μL aliquot of sample solution was dropped onto freshly cleaved mica substrate followed by air drying prior to observation. Afterwards, the topographies of LBP fractions were obtained with an AFM (XE-70, Park Scientific Instruments, Suwon, Korea) with tapping mode in air at room temperature (humidity: 50–60%). Nanoscope Analysis software 1.8 (Bruker, Berlin, Germany) was used for image manipulation.

### 3.7. FT-IR Spectra

FT-IR analysis was applied to obtain IR spectra of the single-extracted samples using a Nicolet iN10 instrument (Thermo Fisher Scientific, Waltham, MA, USA). The dried polysaccharide samples (2 mg) were mixed with KBr powder, ground and then pressed into the pellets for FT-IR scanning in the frequency range of 4000–400 cm^−1^. The data obtained were analyzed using Origin Pro 9.1 software (OriginLab, Northampton, MA, USA).

### 3.8. Nuclear Magnetic Resonance Spectroscopy (NMR)

For NMR analysis, LBP samples (10 mg) were dehydrated twice with 500 µL of D_2_O (99.96%) using a lyophilizer (Christ, Alpha 1-4 LDplus, Osterode, Germany) before final dissolution in 500 µL of high-quality D_2_O. The ^1^H-NMR spectra were collected using a DD2-600 spectrometer (Agilent, Santa Clara, CA, USA) at room temperature.

## 4. Conclusions

In the present study, water-, acid- and alkali-extraction of LBPs were compared systematically. After pretreatment and extraction, nine kinds of polysaccharides, AL_L_, AL_H_, A_L_, A_H_, W_H_, W_H_AL_H_, W_H_AL_L_, A_H_AL_H_, A_L_AL_L_, were obtained. Conclusions for each extraction method were summarized in Table 3. The monosaccharide analysis indicated that AL_L_ consists mainly of RG-I structure with highly branched side chains, while acidic-extracted had more HG regions with less of a substantial part of the side chain, particularly arabinan. W_H_ was a kind of acidic heteropolysaccharide. SEC-MALLS and AFM confirmed large-size polymers with branched morphologies in alkali-extracted polysaccharides. FT-IR and NMR spectra showed that both AL_L_ and AL_H_ were non-esterified polysaccharides and the type of glycosidic bond of all LBPs was mainly β-glycosidic. Further work is required to purify and fractionate the crude polysaccharides to identify the fine structure.

## Figures and Tables

**Table 1:** Monosaccharide composition, monosaccharide ratio, protein content, total sugar and yield of LBPs extracted by different methods.

Sample	Yield	Mol									Rha/GalA	(Ara + Gal)/Rha	Total Sugar (%)	Total Protein (%)
		Man	Rib	Rha	GlcA	GalA	Glc	Gal	Ara	Fuc				
W_H_	1.79 ± 0.16	2.79 ± 0.09	0.57 ± 0.16	4.17 ± 0.29	1.64 ± 0.10	19.75 ± 0.05	4.59 ± 0.04	20.74 ± 0.23	39.13 ± 0.06	6.62 ± 0.03	0.211	14.36	56.68 ± 5.14	7.46 ± 1.13
A_H_	2.50 ± 0.07	3.78 ± 0.08	1.00 ± 0.54	9.88 ± 0.04	3.84 ± 1.66	23.67 ± 0.70	3.44 ± 0.12	24.11 ± 0.04	24.31 ± 0.03	5.37 ± 0.06	0.417	4.9	46.70 ± 6.51	15.76 ± 1.36
AL_H_	4.18 ± 0.05	7.39 ± 0.05	0.70 ± 0.13	8.21 ± 0.12	2.60 ± 0.16	6.34 ± 0.08	4.14 ± 0.04	25.23 ± 0.12	29.17 ± 0.04	16.22 ± 0.03	1.295	6.63	53.34 ± 0.05	33.48 ± 2.37
A_L_	1.80 ± 0.09	2.24 ± 0.01	ND	7.47 ± 0.10	1.84 ± 0.02	28.24 ± 0.05	9.06 ± 0.01	20.54 ± 0.03	22.94 ± 0.06	7.68 ± 0.04	0.264	5.82	40.71 ± 0.03	6.48 ± 1.08
AL_L_	2.46 ± 0.11	4.11 ± 0.17	ND	7.66 ± 0.03	1.61 ± 0.19	14.53 ± 0.13	6.18 ± 0.03	22.23 ± 0.08	37.26 ± 0.09	6.43 ± 0.28	0.527	7.77	58.11 ± 6.99	35.30 ± 1.76
W_H_AL_H_	2.04 ± 0.10	ND	ND	16.27 ± 0.33	ND	13.30 ± 0.20	2.65 ± 0.13	25.65 ± 0.33	37.15 ± 0.02	4.98 ± 0.06	1.223	3.86	46.04 ± 4.63	37.11 ± 2.13
W_H_AL_L_	0.89 ± 0.07	2.67 ± 0.03	ND	11.99 ± 0.24	0.88 ± 0.11	22.08 ± 0.06	4.19 ± 0.08	12.81 ± 0.03	43.89 ± 0.04	1.52 ± 0.13	0.543	4.73	38.61 ± 1.23	29.68 ± 1.39
A_H_AL_H_	1.14 ± 0.07	6.98 ± 1.88	1.02 ± 0.67	13.9 ± 0.09	4.01 ± 0.02	25.46 ± 0.12	3.38 ± 0.07	22.25 ± 0.13	17.73 ± 0.03	5.27 ± 0.02	0.546	2.88	56.44 ± 6.04	24.55 ± 1.18
A_L_AL_L_	0.92 ± 0.03	2.06 ± 0.13	ND	11.04 ± 0.10	1.23 ± 0.26	31.06 ± 0.17	5.33 ± 0.19	13.44 ± 0.13	31.84 ± 0.19	4.00 ± 0.32	0.355	4.1	55.49 ± 4.26	28.73 ± 1.26

W_H_: water extraction at high temperature at 85 °C for 3 h; A_H_: acid extraction at high temperature at 85 °C for 3 h; AL_H_: alkali extraction at high temperature at 85 °C for 3 h; A_L_: acid extraction at low temperature at 28 °C for 40 min; AL_L_: alkali extraction at low temperature at 32 °C for 10 min; W_H_AL_H_: alkali extraction of residue after water extraction (both at 85 °C for 3 h); W_H_AL_L_: alkali extraction at low temperature (32 °C for 10 min) after high-temperature water extraction (85 °C for 3 h); A_H_AL_H_: alkali extraction of residue after acid extraction (both at 85 °C for 3 h); A_L_AL_L_: alkali extraction (32 °C for 10 min) of residue after acid extraction (28 °C for 40 min); ND: not detected; values are mean ± SD. Man: mannose; Rib: ribose; Rha: rhamnose; GlcA: glucuronic acid; GalA: galacturonic acid; Glc: glucose; Gal: galactose; Ara: arabinose; Fuc: fucose.

**Table 2:** Average values of molecular weight and radius of LBPs.

	Mw ^a^ (kDa)		Mn ^b^ (kDa)		Mw/Mn		Rz ^c^ (nm)	
Sample	Fraction I	Fraction II	Fraction I	Fraction II	Fraction I	Fraction II	Fraction I	Fraction II
W_H_	49.44 ± 0.590%	3.078 ± 3.549%	29.51 ± 0.785%	2.432 ± 1.906%	1.675 ± 0.982%	1.266 ± 2.456%	62.0 ± 0.7%	68.8 ± 1.5%
A_H_	199.2 ± 0.914%	3.954 ± 4.782%	150.4 ± 1.104%	3.355 ± 1.986%	1.145 ± 1.457%	1.178 ± 2.668%	62.4 ± 1.3%	71.5 ± 2.1%
AL_H_	223.1 ± 1.803%	26.32 ± 3.206%	130.9 ± 1.393%	15.84 ± 2.424%	1.704 ± 1.825%	1.662 ± 3.301%	52.0 ± 1.2%	57.6 ± 1.9%
A_L_	2334 ± 2.481%	717.4 ± 3.313%	1690 ± 3.738%	679.6 ± 3.269%	1.381 ± 4.486%	1.056 ± 4.654%	59.3 ± 3.0%	68.2 ± 2.4%
AL_L_	7162 ± 0.686%	1582 ± 2.061%	2539 ± 1.072%	1495 ± 2.082%	2.821 ± 1.273%	1.058 ± 2.930%	56.6 ± 0.6%	64.8 ± 1.3%
W_H_AL_H_	207.1 ± 1.182%	18.27 ± 2.720%	112.6 ± 2.529%	13.82 ± 2.544%	1.839 ± 1.653%	1.322 ± 2.567%	53.1 ± 2.1%	66.1 ± 1.7%
W_H_AL_L_	6371 ± 0.684%	1239 ± 1.779%	2289 ± 0.506%	1037 ± 0.919%	2.783 ± 0.771%	1.195 ± 1.240%	55.1 ± 0.7%	64.4 ± 0.7%
A_H_AL_H_	194.6 ± 1.487%	29.68 ± 2.960%	105.7 ± 2.714%	18.01 ± 2.513%	1.841 ± 1.507%	1.648 ± 2.519%	47.4 ± 2.1%	58.3 ± 3.0%
A_L_AL_L_	2294 ± 3.384%	1035 ± 3.589%	1062 ± 2.529%	952.1 ± 3.283%	2.160 ± 2.854%	1.087 ± 3.281%	53.4 ± 1.5%	69.6 ± 2.3%

W_H_: water extraction at high temperature at 85 °C for 3 h; A_H_: acid extraction at high temperature at 85 °C for 3 h; AL_H_: alkali extraction at high temperature at 85 °C for 3 h; A_L_: acid extraction at low temperature at 28 °C for 40 min; AL_L_: alkali extraction at low temperature at 32 °C for 10 min; W_H_AL_H_: alkali extraction of residue after water extraction (both at 85 °C for 3 h); W_H_AL_L_: alkali extraction at low temperature (32 °C for 10 min) after high-temperature water extraction (85 °C for 3 h); A_H_AL_H_: alkali extraction of residue after acid extraction (both at 85 °C for 3 h); A_L_AL_L_: alkali extraction (32 °C for 10 min) of residue after acid extraction (28 °C for 40 min). ^a^ Mw: weight-average of Molar mass. ^b^ Mn: number-average of molar mass. ^c^ Rz: z-average of root mean square radius of gyration.

**Table 3:** Conclusions for each extraction method.

Samples	Conclusion
W_H_	The free heteropolysaccharide in Goji cell wall could be extracted by hot water
A_H_	High-temperature-acid reserved more HG regions and degraded the side chains
A_L_	Low-temperature-acid reserved not only more HG regions, but also RG-I and side chains
AL_H_	HG regions was destroyed in severe alkaline condition
AL_L_	More extensively branched RG-I domains
W_H_AL_H_	The polysaccharides released by hot alkali were difficult to be extracted by hot water, and the yield was still high
W_H_AL_L_	Hot water extracted free heteropolysaccharide, then more RG-I, relatively, were extracted by mild alkali
A_H_AL_H_	The polysaccharides released by hot alkali were difficult to be extracted by hot acid, and the yield was still high
A_L_AL_L_	The RG-I extracted by mild alkali were difficult to be extracted by mild acid
